# Supplementary material for: Intratumoral IL-12 delivery empowers CAR-T cell immunotherapy in a pre-clinical model of glioblastoma
Source: Nat Commun. 2021 Jan 19;12:444. doi: 10.1038/s41467-020-20599-x (PMC7815781; doi:10.1038/s41467-020-20599-x)
Supplement: Supplementary file 1 — Supplementary Information [file 41467_2020_20599_MOESM1_ESM.pdf]

SUPPLEMENTARY INFORMATION

Intratumoral IL-12 delivery empowers CAR-T cell immunotherapy in a pre-clinical model of glioblastoma

Giulia Agliardi, Anna Rita Liuzzi et al.

Corresponding authors:
Burkhard Becher. E-Mail: becher@immunology.uzh.ch
Karin Straathof. E-Mail: k.straathof@ucl.ac.uk

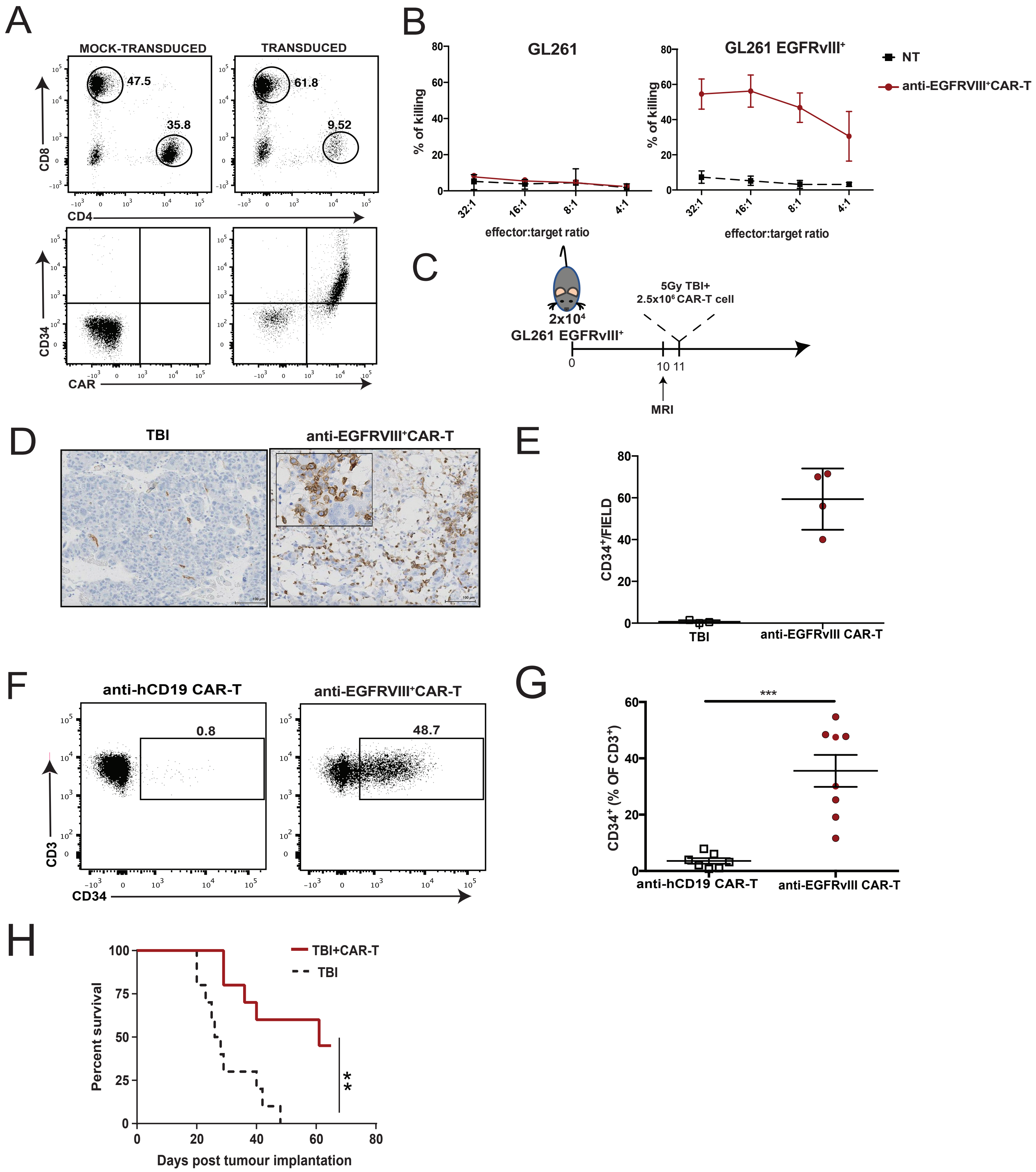

Supplementary Figure 1. Murine CAR-T cells recognize EGFRvIII+ GL261 in vitro and specifically infiltrate EGFRvIII+ tumors in vivo. **(A)** Transduction efficiency of mock-transduced splenocytes (left panel) and EGFRvIII CAR-transduced splenocytes (right panel). Transduced cells co-express CD34 (y axis) and the CAR (x axis) (stained with an EGFRvIII peptide conjugated to mouse IgG2a). **(B)** Chromium release assay showing specific lysis of EGFRvIII-expressing GL261 but not parental cell line GL261 by anti-EGFRvIII CAR-T cells (Data are presented as mean ± SD of biological replicates from three independent experiments). **(C)** Experiment timeline. GL261 EGFRvIII+ cells were implanted in the right striatum at day 0. Tumor engraftment was confirmed at day 10 by MRI and the day after mice received 5Gy total body irradiation followed by intravenous injection of 2.5x10^6 CAR-T cells. Mice receiving TBI only but no T cells were used as control. **(D)** CAR-T cell infiltration 7 days post infusion was confirmed by immunohistochemistry for the marker gene CD34. Mice receiving TBI but no T cells were used as control. **(E)** Quantification of **(D)** (Data are presented as mean values ± SD n=4 mice. **(F)** CAR-T cell antigen-specific infiltration was confirmed by FACS analysis 8 days post T cell transfer. CAR-T cells were identified as CD3+CD34+ cells and hCD19-specific CAR T cells were used as negative control **(G)** Quantification of **(F)**: CAR-T cell infiltration expressed as percentage of CD34+ T cells of total CD3+ (Data are presented as mean values ± SEM. hCD19 CAR n=7, EGFRvIII CAR n=8 biological replicates from 2 independent experiments). **(H)** Survival curves of **(C)** p=0.0028 (**), Log-rank test, n=10 per group from two independent experiments. Source data are provided as a Source Data file.

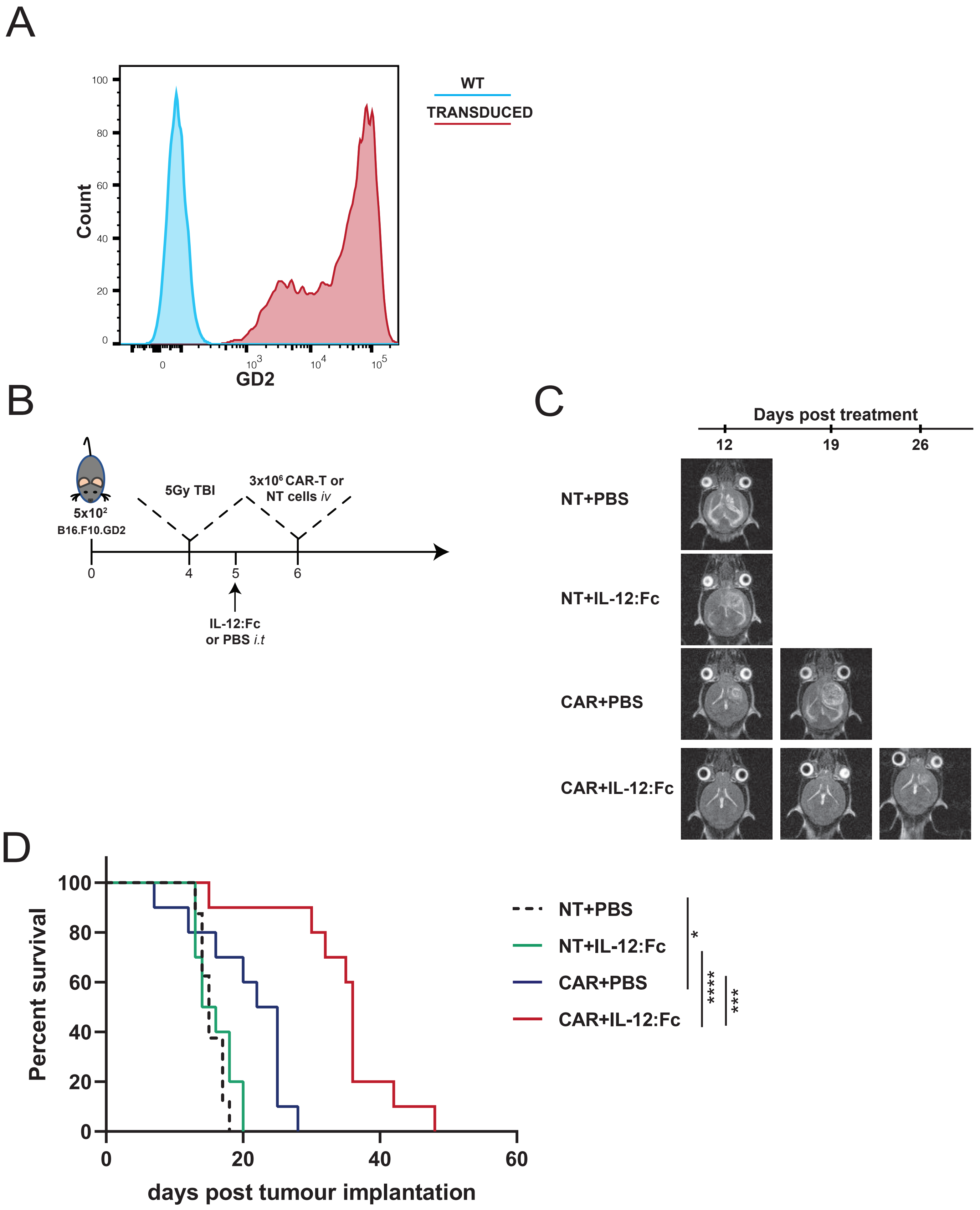

Supplementary Figure 2. Combination of GD2-specific CAR-T cells and IL-12:Fc enables better control of intracranial B16.F10.GD2+ tumors. (A) B16.F10 cells were transduced to express the GD2/GD3 synthase and stained for surface GD2 expression (Blue line: wild type; Red line: transduced). **(B)** Experiment timeline. B16.F10.GD2+ cells were implanted in the right striatum at day 0. Mice received 5Gy TBI on day 4 post implantation, followed by either PBS or 300ng of IL-12:Fc at tumor site on day 5, and intravenous injection of $3x10^6$ CAR-T cells or non-transduced cells. Tumor growth was monitored weekly. **(C)** Representative MRI images (axial view) of one mouse per group. **(D)** Survival curves (NT+PBS n=8, CAR+PBS n=10, NT+IL-12:Fc n=10, CAR+IL-12:Fc n=10 treated in two independent experiments) are shown. * p=0.0108, *** =0.0001, ****p<0.0001 (Log-rank test). Source data are provided as a Source Data file.

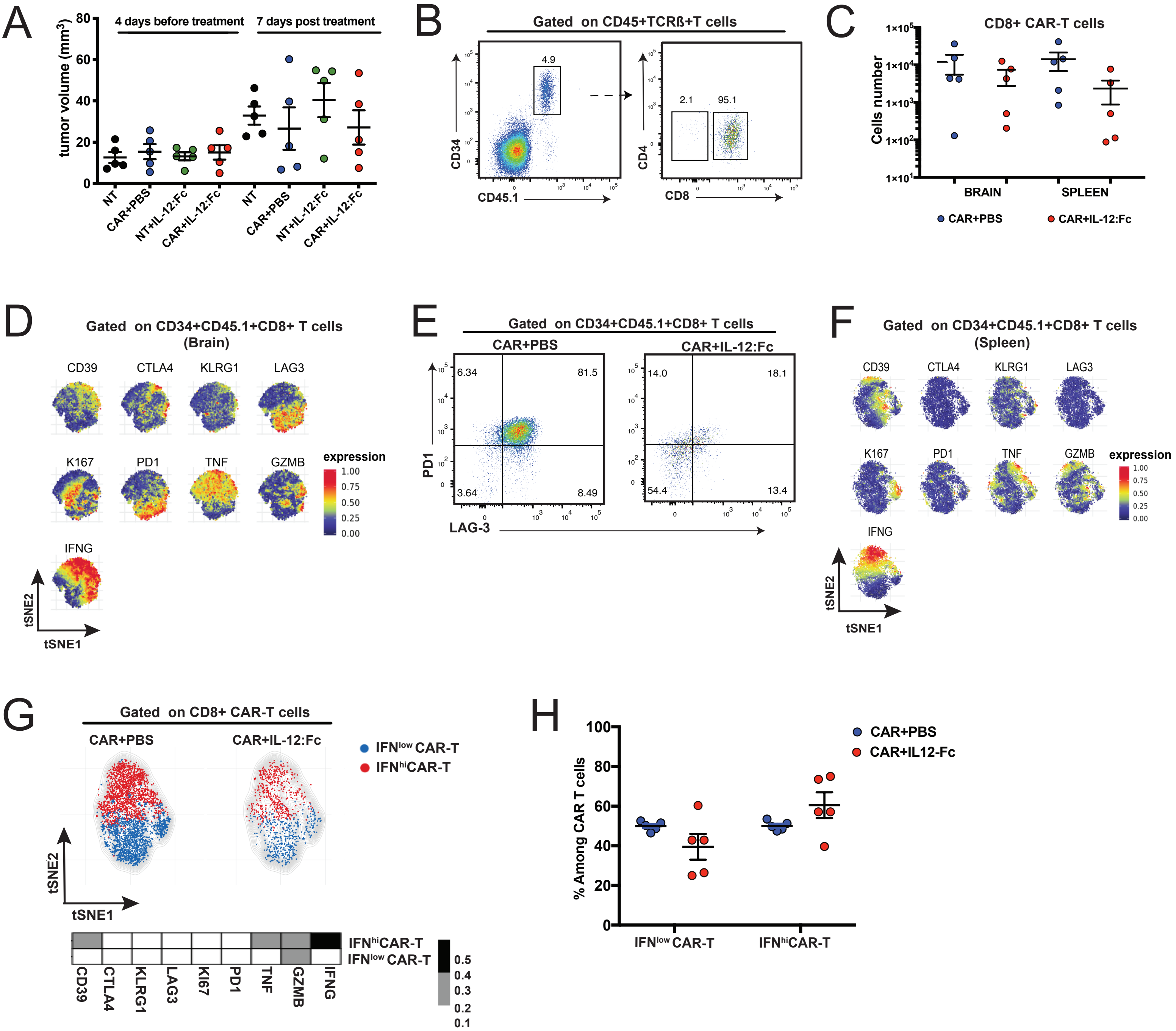

Supplementary Figure 3. IL-12:Fc administration does not affect CAR-T cell infiltration within the tumor, but improves their pro-inflammatory phenotype. (A) Tumor volume per each condition at day 4 before treatment and day 7 post treatment, (NT+PBS n=4, NT+IL-12:Fc n=5, CAR+PBS n=5, CAR+IL-12:Fc n=5 mice, representative of n=2 independent experiments). **(B)** Manual gating of flow cytometry data on CAR T defined as CD45.1+TCR-beta+CD34+ and afterwards divided in CD4 and CD8 positive cells. Representative brain sample among glioma-bearing mice treated with CAR+IL-12:Fc. **(C)** Cell number of CD8+ CAR T cells in brain and spleen in mice receiving either CAR-T cells alone or in combination with IL-12:Fc, n=5 mice per condition from one experiment. **(D)** t-SNE map displaying stochastically selected CAR T-cells CD34+CD45.1+ CD8+ T cells from brain. **(E)** Manual gating of flow cytometry data on CAR T cells CD8+ LAG3+PD1+ in mice receiving either CAR T cells alone or in combination with IL-12:Fc. **(F)** t-SNE map displaying stochastically CAR-T cells CD34+CD45.1+ CD8+ T cells from spleen. **(G)** t-SNE map showing the FlowSOM-guided metaclustering gated on CD34+CD45.1+ CD8+CAR T-cells from spleen and heatmap showing the median marker expression for each defined metacluster (value range: 0–1). **(H)** Frequencies of the two CD34+CD45.1+CD8+CAR-T cells subclusters among total CD8+CAR T-cells within the different conditions in the spleen, n=5 mice per condition. Data are presented as mean values ± SEM. Ordinary One-way Anova with Dunnett multiple comparison test **(A)**, 2-tailed Unpaired Mann-Whitney T test **(C, H)**. Source data are provided as a Source Data file.

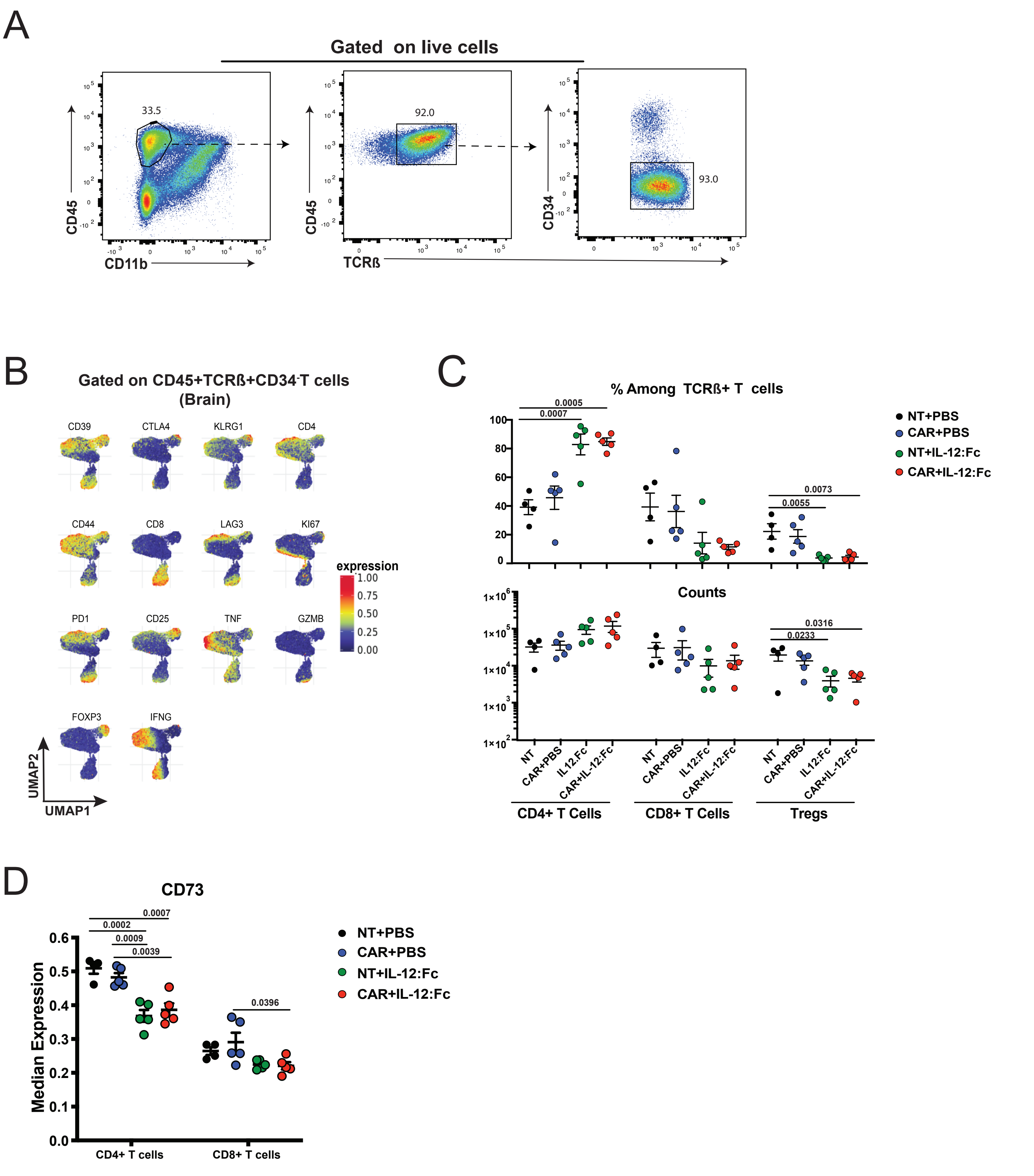

Supplementary Figure 4. Post treatment endogenous T cells frequency and functional marker median expression. (A) Manual gating for endogenous T cells: live cells were gated on CD45+CD11b+ cells, T cells were defined as CD45+TCR-beta+ cells and subsequently as TCR-beta+CD34- cells to exclude CAR-T cells. Representative sample among glioma-bearing mice treated with CAR-T cells. **(B)** UMAP displaying stochastically selected CD45+TCR-β+ T cells from brain. **(C)** Frequencies (top panel) and cell counts (bottom panel) of the three TCR-β+ T-cells subclusters among total TCR-β+ T-cells within the different conditions, (NT+PBS n=4, NT+IL-12:Fc n=5, CAR+PBS n=5, CAR+IL-12:Fc n=5 mice, replicates from 2 independent experiments). **(D)** CD73 median expression on CD4 and CD8 T cells, (NT+PBS n=4, NT+IL-12:Fc n=5, CAR+PBS n=5, CAR+IL-12:Fc n=5, replicates from 2 independent experiments). Data are presented as mean values ± SEM. Ordinary One-way Anova with Dunnett's multiple comparison **(C)**, Ordinary One-way Anova with Turkey's multiple comparison **(D)**. Source data are provided as a Source Data file.

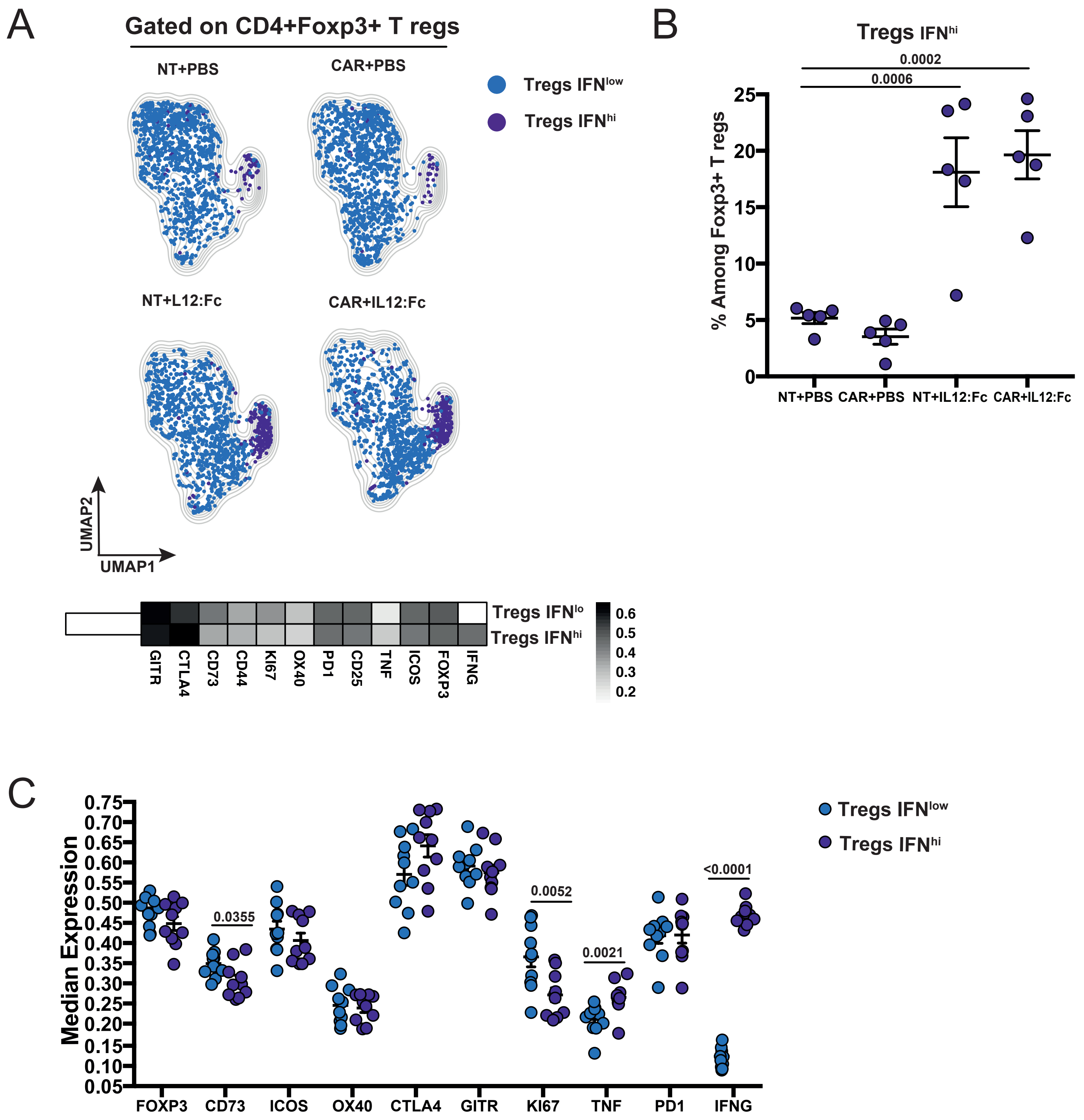

Supplementary Figure 5. IL-12:Fc administration increases the frequency of IFN-gamma producing Treg. (A) UMAP displaying stochastically selected CD4+Foxp3+ Tregs exported from live TCR-β+ T-cells. Heatmap showing the median marker expression for each defined metacluster (value range: 0–1). **(B)** Frequency of IFN-gamma high Tregs in each condition, n=5 mice per condition. **(C)** Median expression of selected cell markers shown for Tregs with high (IFN-gamma hi) and low (IFN-gamma low) IFN-gamma production in both IL-12:Fc and CAR+IL-12:Fc conditions, n=10 mice per cluster. Data are presented as mean values ± SEM. Ordinary One-way Anova with Dunnett's multiple comparison **(B)**, 2-tailed Unpaired Mann-Whitney T test **(C)**. Representative of 2 independent experiments. Source data are provided as a Source Data file.

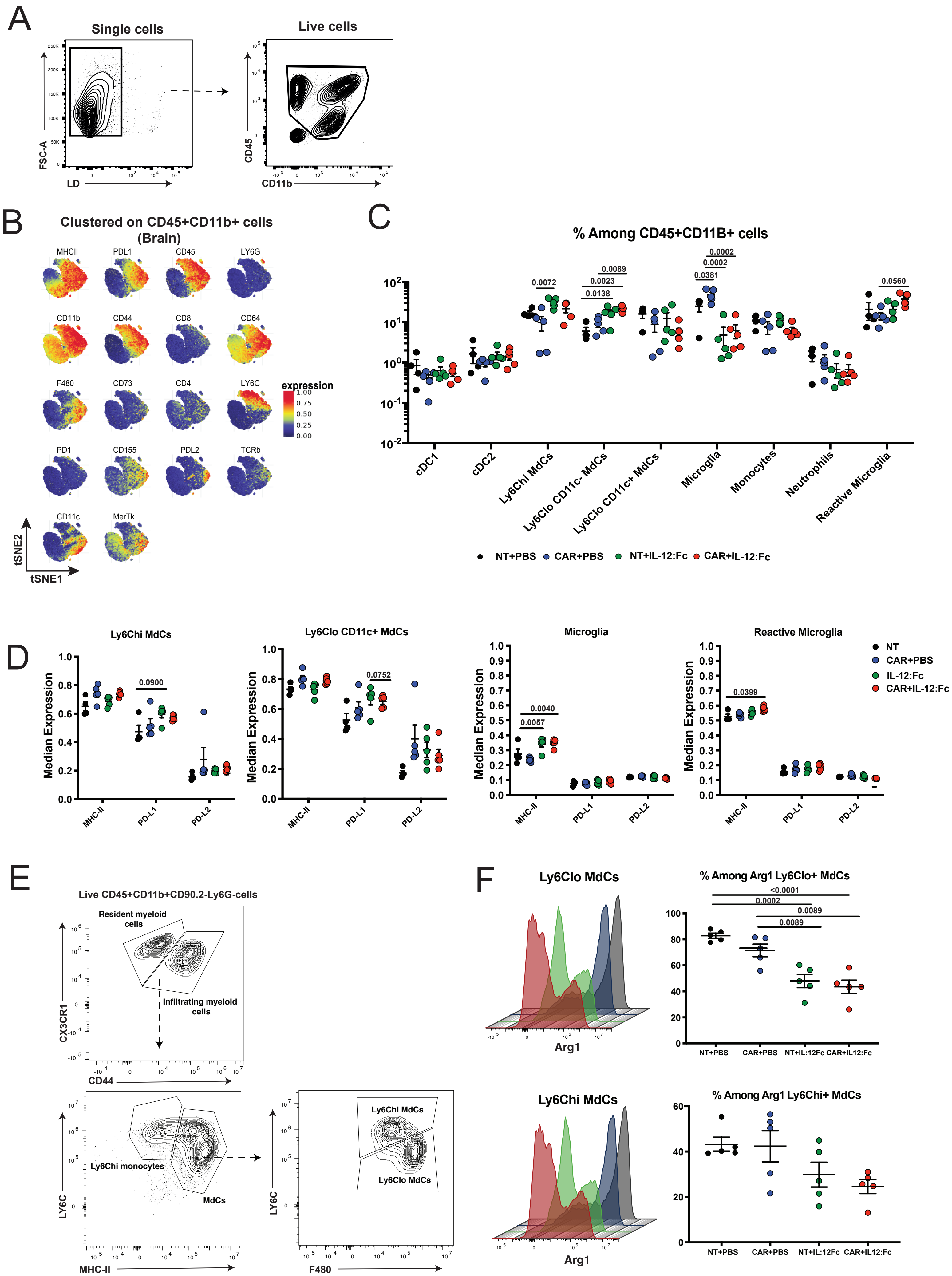
A
Single cells
FSC-A
LD
Live cells
CD45
CD11b
B
Clustered on CD45+CD11b+ cells (Brain)
MHCII
PDL1
CD45
LY6G
CD11b
CD44
CD8
CD64
F480
CD73
CD4
LY6C
expression
1.00
0.75
0.50
0.25
0.00
PD1
CD155
PDL2
TCRb
CD11c
MerTk
tSNE2
tSNE1
C
% Among CD45+CD11B+ cells
0.0072
0.0089
0.0023
0.0138
0.0002
0.0002
0.0381
0.0560
cDC1
cDC2
Ly6Chi MdCs
Ly6Clo CD11c- MdCs
Ly6Clo CD11c+ MdCs
Microglia
Monocytes
Neutrophils
Reactive Microglia
NT+PBS
CAR+PBS
NT+IL-12:Fc
CAR+IL-12:Fc
D
Ly6Chi MdCs
0.0900
Median Expression
MHC-II
PD-L1
PD-L2
Ly6Clo CD11c+ MdCs
0.0752
Microglia
0.0040
0.0057
Reactive Microglia
0.0399
NT
CAR+PBS
IL-12:Fc
CAR+IL-12:Fc
E
Live CD45+CD11b+CD90.2-Ly6G-cells
Resident myeloid cells
Infiltrating myeloid cells
CX3CR1
CD44
Ly6Chi monocytes
MdCs
LY6C
MHC-II
Ly6Chi MdCs
Ly6Clo MdCs
F480
F
Ly6Clo MdCs
Arg1
% Among Arg1 Ly6Clo+ MdCs
<0.0001
0.0002
0.0089
0.0089
NT+PBS
CAR+PBS
NT+IL:12Fc
CAR+IL12:Fc
Ly6Chi MdCs
Arg1
% Among Arg1 Ly6Chi+ MdCs
NT+PBS
CAR+PBS
NT+IL:12Fc
CAR+IL12:Fc

Supplementary Figure 6. Post treatment endogenous myeloid cells frequency and functional marker median expression.
(A) Manual gating of flow cytometry data on major leukocyte populations present in a representative brain sample of glioma. Live cells were gated on CD11b+CD45+ cells and exported in R for FlowSOM metaclustering. CD45+CD11b- cells were excluded after the metaclustering. **(B)** Umap displaying stochastically selected CD45+CD11b+ cells from brain. **(C)** Frequencies of the nine CD11b+CD45+ subclusters among total CD11b+CD45+ cells within the different conditions, (NT+PBS n=4, NT+IL-12:Fc n=5, CAR+PBS n=5, CAR+IL-12:Fc n=5 mice, replicates from 2 independent experiments). **(D)** Median expression of selected cell markers shown for each condition on Ly6Chi MdCs, Ly6Clo CD11c+ MdCs, Microglia and Reactive Microglia, (NT+PBS n=4, NT+IL-12:Fc n=5, CAR+PBS n=5, CAR+IL-12:Fc n=5 mice, replicates from 2 independent experiments). **(E)** Manual gating for MdCs: live cells were gated on CD45+CD11b+CD90.2-Ly6G-cells, next CD44+CX3CR1+ cells were defined as infiltrating myeloid cells and subsequently Ly6C+MHC-II+ cells were defined as MdCs. MdCs were further characterized as F480+and by the amount of expression of Ly6C. **(F)** Frequency of Arg1 positive MdCs Ly6Clow (upper panel) and Ly6Chigh (lower panel), n=5 mice per condition from one experiment. Data are presented as mean values ± SEM. Ordinary One-way Anova with Turkey's multiple comparison **(C-D, F).** Source data are provided as a Source Data file.

.

Supplementary Table 1. List of antibodies.

Extracellular staining

Antigens	Fluorochromes	Host	Isotypes	Clones	Manufacturers	Dilution Factor	Catalogoue Number
**CD103**	Alexa488	armenian hamster	IgG	2E7	eBioscience	100	11103185
**CD11b**	BUV661	rat	IgG2b	M1/70	BD PharmigenTM	400	565080
**CD11b**	BUV737	rat	IgG2b	M1/70	BD	400	564443
**CD11c**	PE-Cy5.5	armenian hamster	IgG	N418	eBioscience	400	35-0114-82
**CD155**	PE	rat	IgG2a	TX56	BioLegendTM	100	131507
**CD160**	PE	rat	IgG2a	7H1	BioLegendTM	50	143003
**CD206**	Alexa700	rat	IgG2a	C068C2	BioLegendTM	100	141734
**CD25**	BV650	rat	IgG1	PC61	BioLegendTM	100	102038
**CD27**	BV480	mouse	IgG1	LG3A10	BD PharmigenTM	200	746742
**CD3**	BV785	rat	IgG2b, k	17A2	Biolegend	100	100232
**CD34**	FITC	rat	IgG2a	RAM34	eBioscience	100	11-0341-82
**CD39**	A647	rat	IgG2a	Duha59	BioLegendTM	400	143807
**CD39**	PerCP-efluor710	rat	IgG2b	24DMS1	eBioscience	800	46-0391-80
**CD4**	BV650	mouse	IgG2a	RM4-5	BioLegendTM	400	100546
**CD4**	BUV496	rat	IgG2b	GK1.5	BD PharmigenTM	100	564667
**CD44**	BUV737	rat	IgG2b	IM7	BD PharmigenTM	200	612799
**CD44**	BV650	rat	IgG2b	IM7	BioLegend	200	103049
**CD45**	BUV395	mouse	IgG2b	30-F11	BD PharmigenTM	400	564279
**CD45**	BUV563	rat	IgG2b	30-F11	BD PharmigenTM	400	565710
**CD45.1**	BV570	mouse	IgG2a	A20	BioLegendTM	200	110733
**CD45.1**	Biotin	mouse	IgG2a	A20	BioLegendTM	200	103103
**CD45.1**	BV785	mouse	IgG2a	A20	BioLegendTM	100	110743
**CD45.2**	Pacific Blue	mouse	IgG2a	104	BioLegend	400	110722
**CD64**	BV421	mouse	IgG1	X54-5/7.1	BioLegendTM	100	139309
**CD64**	PE	mouse	IgG1	X54-5/7.1	BioLegendTM	100	139304
**CD73**	BV605	rat	IgG1	TY/11.8	BioLegendTM	100	127215
**CD73**	APC-Cy7	rat	IgG1	TY/11.8	BioLegendTM	100	127232
**CD8**	BUV805	rat	IgG2a	53-6.7	BD PharmigenTM	100	564920
**CX3CR1**	BV605	mouse	IgG2a	SA011F11	BioLegend	400	149027
**EGFRvIII**	Purified	mouse	IgG2a	MR1.1	In house	500 ng/sample	
**EGFRvIII CAR**	AF488			EGFRvIII:mIgG2a	In house	500 ng/sample	
**F4/80**	BV510	rat	IgG2a	BM8	BioLegend	100	123135
**F4/80**	PE-Cy5	rat	IgG2a	BM8	BioLegend	400	123112
**GD2**	PE	Mouse	IgG2a, κ	14G2a	BioLegendTM	200	357303
**GITR**	FITC	rat	IgG2b	DTA-1	BioLegendTM	1600	126308
**ICOS**	PE	rat	IgG2b, k	7E.17G9	BioLegendTM	100	117406
**KLRG-1**	APC-C7	syrian hamster	IgG	2F1/KLRG1	BioLegendTM	100	138426
**LAG3**	BV421	rat	IgG1	C9B7W	BioLegendTM	100	125221
**Ly-6C**	BV711	rat	IgG2c	HK1.4	BioLegendTM	400	128037
**LAP**	BV421	mouse	IgG1	TW7-16B4	BioLegend	100	141407
**Ly6G**	BUV563	rat	IgG2a	1A8	BD PharmigenTM	200	565707
**Ly6G**	FITC	rat	IgG2a	1A8	BioLegend		127606
**MerTK**	PE-Cy7	rat	IgG2a	DS5MMER	eBioscience	100	25-5751-82
**MerTK**	SuperBright 780	rat	IgG2a	DS5MMER	eBioscience	100	78-5751-82
**MHC-II**	BB700	rat	IgG2b	M5/114.15.2	BD	400	746197
**NK1.1**	BV785	mouse	IgG2a	PK136	BioLegendTM	100	108749
**OX40**	Biotin	rat	IgG1	OX-86	BioLegendTM	100	119403
**PD-1**	BV785	rat	IgG2a	29F.1A12	BioLegendTM	100	135225
**PD-1**	BV605	rat	IgG2a	29F.1A12	BioLegendTM	100	135220
**PD-L1**	APC	rat	IgG2b	10F.9G2	BioLegendTM	150	124311
**PD-L1**	PE-Cy7	rat	IgG2a	MIH5	eBioscience	200	25-5982-82
**PD-L2**	PE-Dazzle594	rat	IgG2a	TY25	BioLegendTM	150	107216
**Streptavidin**	BUV395				BD PharmigenTM	400	564176
**Streptavidin**	BV570				BioLegend	400	405227
**TCRb**	PE-Cy5	armenian hamster	IgG	H57-597	BioLegendTM	400	109209
**XCR-1**	Alexa647	mouse	*IgG2b*	ZET	BioLegendTM	200	148213

**LIVE/DEAD fixable Acqua dead cell stains**			ThermoFisher
**LIVE/DEAD fixable Near-IR dead cell stains**			ThermoFisher

Intracellular staining

Antigens	Fluorochromes	Host	Isotypes	Clones	Manufacturers	Dilution Factor	Catalogoue Number
**Arginase-1**	APC	rat	IgG2a, k	A1exF5	Invitrogen	200	17-3697-82
**CTLA-4**	Alexa700	armenian hamster	IgG1	UC10-4F10-11	BD PharmigenTM	600	565778
**Eomes**	PerCP-eFluor710	rat	IgG2a	Dan11mag	eBioscience	150	61-4875-82
**FOXP3**	PE-eFlour610	rat	IgG2a	FJK-16s	eBioscience	200	61-5773-82
**GRANZYME**	PE	mouse	IgG1	GB11	BD PharmigenTM	200	561142
**IFN-γ**	PE-Cy7	rat	IgG1	XMG1.2	eBioscience	400	25-7311-82
**IFN-γ**	APC	rat	IgG1	XMG1.2	BioLegendTM	400	505810
**IL10**	PE-Dazzle594	rat	IgG2b	JES5-16E3	BioLegend	100	505033
**KI-67**	BV480	mouse	IgG1	B56	BD PharmigenTM	100	566109
**TNF**	BV711	rat	IgG	MP6-XT22	BioLegendTM	200	506349

Secondary Ab

Antigens	Fluorochromes	Host	Isotypes	Clones	Manufacturers	Dilution Factor	Catalogoue Number
**mIgG2a**	PE	Goat		polyclonal	Jackson	1000	115-115-206-JIR

Immunohistochemistry Ab

Antigens	Fluorochromes	Host	Isotypes	Clones	Manufacturers	Dilution Factor	Catalogoue Number
**EGFRvIII**	Purified	Mouse	IgG1	L84A	Absolute Antibody	1000	Ab00184-1.1
**CD34**	Purified	Rat	IgG2A, k	RAM34	eBioscience	100	14-0341-82
