# Supplementary material for: Intratumoral IL-12 delivery empowers CAR-T cell immunotherapy in a pre-clinical model of glioblastoma
Source: Nat Commun. 2021 Jan 19;12:444. doi: 10.1038/s41467-020-20599-x (PMC7815781; doi:10.1038/s41467-020-20599-x)
Supplement: Supplementary file 3 — Reporting Summary [file 41467_2020_20599_MOESM3_ESM.pdf]

nature research

Corresponding author(s): Karin Straathof & Burkhard Becher

Last updated by author(s): 20/11/20

Reporting Summary

Nature Research wishes to improve the reproducibility of the work that we publish. This form provides structure for consistency and transparency in reporting. For further information on Nature Research policies, see our Editorial Policies and the Editorial Policy Checklist.

Statistics

For all statistical analyses, confirm that the following items are present in the figure legend, table legend, main text, or Methods section.

n/a	Confirmed	
☐	☒	The exact sample size (*n*) for each experimental group/condition, given as a discrete number and unit of measurement
☐	☒	A statement on whether measurements were taken from distinct samples or whether the same sample was measured repeatedly
☐	☒	The statistical test(s) used AND whether they are one- or two-sided<br>*Only common tests should be described solely by name; describe more complex techniques in the Methods section.*
☒	☐	A description of all covariates tested
☐	☒	A description of any assumptions or corrections, such as tests of normality and adjustment for multiple comparisons
☐	☒	A full description of the statistical parameters including central tendency (e.g. means) or other basic estimates (e.g. regression coefficient) AND variation (e.g. standard deviation) or associated estimates of uncertainty (e.g. confidence intervals)
☐	☒	For null hypothesis testing, the test statistic (e.g. *F*, *t*, *r*) with confidence intervals, effect sizes, degrees of freedom and *P* value noted<br>*Give P values as exact values whenever suitable.*
☒	☐	For Bayesian analysis, information on the choice of priors and Markov chain Monte Carlo settings
☒	☐	For hierarchical and complex designs, identification of the appropriate level for tests and full reporting of outcomes
☒	☐	Estimates of effect sizes (e.g. Cohen's *d*, Pearson's *r*), indicating how they were calculated

Our web collection on statistics for biologists contains articles on many of the points above.

Software and code

Policy information about availability of computer code

Data collection	MRI images were acquired on 1T ICON system (Bruker).<br>Immunohistochemistry images were taken with a Leica DMD108 photographic microscope.<br>Cytokine bead array (legendplex) was acquired on a Fortessa flow cytometer (BD) (FACS DIVA Software v9).<br>After surface and intracellular staining, cells were acquired on a Symphony Flow Cytometer (BD Biosciences, FACS DIVA Software v9) or Aurora spectral flow cytometer (Cytek).
Data analysis	Cytokine bead array were analyzed using Legend Plex software V8.0 (Biolegend).<br>MRI data were exported from Image J (v. 2.0.0-rc-54/1.51h) and analyzed with ITK Snap (v 3.6.0). Graphpad Prism (v6 or 8) was used for statistical analysis.<br>To analyze in details CAR T cells, endogenous T cells, myeloid cells and endogenous T regs, we gated the cluster of interest on FlowJo (version 10.0.8, TreeStar Inc) and then exported for further analyses in RStudio Version 3.6.1 using the script available at: https://github.com/BecherLab/High-dimensional-single-cell-Analysis-for-Cytometry-/blob/master/pipeline_FlowSOM.R. The selection of cofactor for data transformation was checked on Cytobank: https://www.cytobank.org/. Statistical analysis was performed using Graphpad Prism (v8).

For manuscripts utilizing custom algorithms or software that are central to the research but not yet described in published literature, software must be made available to editors and reviewers. We strongly encourage code deposition in a community repository (e.g. GitHub). See the Nature Research guidelines for submitting code & software for further information.

Data

Policy information about availability of data

All manuscripts must include a data availability statement. This statement should provide the following information, where applicable:

- Accession codes, unique identifiers, or web links for publicly available datasets
- A list of figures that have associated raw data
- A description of any restrictions on data availability

The source data are provided as a Source Data file.

The flow cytometry data that support the findings (Figures 3 & 4 and Supplementary Figures 3-6) are available at:https://data.mendeley.com/datasets/xbvcsdp86v/draft.

All other data that support the findings of this study are available from the corresponding authors upon reasonable request

Field-specific reporting

Please select the one below that is the best fit for your research. If you are not sure, read the appropriate sections before making your selection.

☒ Life sciences ☐ Behavioural & social sciences ☐ Ecological, evolutionary & environmental sciences

For a reference copy of the document with all sections, see nature.com/documents/nr-reporting-summary-flat.pdf

Life sciences study design

All studies must disclose on these points even when the disclosure is negative.

Sample size	Assumptions for power analysis were as follows: alpha error: 5%; beta error: 20%. Values for differences in survival were based on pilot experiments.
Data exclusions	No data were excluded
Replication	Experiments were repeated at least twice, unless otherwise specified (refer to figure legends for exact number of repeats).<br>All flow cytometry experiments with multi-functional analysis were performed once with biological replicates or technical replicates (as specified in figure legend).
Randomization	For the GL261_EGFRvIII model, mice were matched into treatment groups according to tumor size (as measured by MRI) at the time of CAR-T cell transfer. Mice with no measurable tumor prior to treatment were excluded. For the B16.F10_GD2 model, mice were randomly assigned to treatment groups as T cells were administered before tumor was detectable by MRI. All mice used in this study were female and 6 to 8 weeks old.
Blinding	Tumor measurements by MRI were performed in a blinded fashion. Objective assessment of the well-being of mice was achieved by the usage of stringent humane endpoints (20% weight loss, hypomotility) and the evaluation by one trained person.

Reporting for specific materials, systems and methods

We require information from authors about some types of materials, experimental systems and methods used in many studies. Here, indicate whether each material, system or method listed is relevant to your study. If you are not sure if a list item applies to your research, read the appropriate section before selecting a response.

Materials & experimental systems

n/a	Involved in the study	
☐	☒	Antibodies
☐	☒	Eukaryotic cell lines
☒	☐	Palaeontology and archaeology
☐	☒	Animals and other organisms
☒	☐	Human research participants
☒	☐	Clinical data
☒	☐	Dual use research of concern

Methods

n/a	Involved in the study	
☒	☐	ChIP-seq
☐	☒	Flow cytometry
☐	☒	MRI-based neuroimaging

Antibodies

Antibodies used

Antigens, Fluorochromes, Host, Isotypes, Clones, Manufacturers, Dilution factor, Catalogue number
CD103, Alexa488, armenian hamster, IgG, 2E7, eBioscience, 100, 11103185
Arginase-1, APC, rat, IgG2a,k, A1exF5, ThermoFisher, 200, 17-3697-82
CD11b, BUV661, rat, IgG2b, M1/70, BD PharmigenTM,400, 565080
CD11b, BUV 737, rat, IgG2b, M1/70, BD, 400, 564443
CD11c, PE-Cy5.5, armenian hamster, IgG, N418, eBioscience,400, 35-0114-82
CD155, PE, rat, IgG2a, TX56, BioLegendTM, 100, 131507
CD160, PE, rat, IgG2a, 7H1, BioLegendTM, 50, 143003
CD206, Alexa700, rat, IgG2a, C068C2, BioLegendTM,100, 141734
CD25, BV650, rat, IgG1, PC61, BioLegendTM, 100, 102038
CD27, BV480, mouse, IgG1, LG3A10, BD, PharmigenTM, 200, 746742
CD3, BV785, 17A2, rat IgG2b, k, 17A2 , Biolegend, 100 100232
CD34, FITC, rat IgG2a, RAM34, eBioscience, 100, 11-0341-82
CD39, A647, rat, IgG2a, Duha59, BioLegendTM, 400, 143807
CD39, PerCP-efluor710,rat, IgG2b, 24DMS1, eBioscience, 800, 46-0391-80
CD4, BV650, mouse, IgG2a, RM4-5, BioLegendTM, 400, 100546
CD4, BUV496, rat, IgG2b, GK1.5, BD PharmigenTM, 100, 564667
CD44, BUV737 rat, IgG2b, IM7, BD PharmigenTM, 200, 612799
CD44, BV 650, rat, IgG2b, IM7, BioLegend, 200, 103049
CD45, BUV395, mouse, IgG2b, 30-F11, BD PharmigenTM, 400, 564279
CD45, BUV563, rat, IgG2b, 30-F11, BD PharmigenTM, 400, 565710
CD45.1, BV570, mouse, IgG2a, A20, BioLegendTM, 200, 110733
CD45.1, Biotin, mouse, IgG2a, A20, BioLegendTM, 200, 103103
CD45.1, BV 785, mouse, IgG2a, A20, BioLegendTM, 100, 110743
CD45.2, Pacific Blue, mouse, IgG2a, 104, BioLegend, 400, 110722
CD64, BV421, mouse, IgG1, X54-5/7.1, BioLegendTM,100, 139309
CD64, PE, mouse, IgG1, X54-5/7.1, BioLegendTM, 100, 139304
CD73, BV605, rat, IgG1, TY/11.8, BioLegendTM, 100, 127215
CD73, APC-Cy7, rat, IgG1, TY/11.8, BioLegendTM, 100, 127232
CD8, BUV805, rat, IgG2a, 53-6.7, BD PharmigenTM, 100, 564920
CTLA-4, Alexa700, armenian hamster, IgG1, UC10-4F10-11, BD PharmigenTM, 600, 565778
CX3CR1, BV 605, mouse, IgG2a, SA011F11, BioLegend,400, 149027
EGFRvIII, purified, MR1.1, 1ug/sample, In house
EGFRvIII CAR, AF488, EGFRvIII:mIgG2a, 500ng/sample In house
Eomes,PerCP-eFluor710, rat, IgG2a, Dan11mag, eBioscience, 150, 61-4875-82
F4/80, BV510, rat, IgG2a, BM8, BioLegendTM, 100, 123135
F4/80, PE-Cy5, rat, IgG2a, BM8, BioLegend, 400, 123112
FOXP3, PE-eFlour610, rat, IgG2a, FJK-16s, eBioscience, 200, 61-5773-82
GD2, PE, Mouse, IgG2a, κ, 14G2a, 200, Biolegend, 357303
GITR, FITC, rat, IgG2b, DTA-1, BioLegendTM, 1600, 126308
GRANZYME, PE, mouse, IgG1, GB11, BD PharmigenTM, 200, 561142
ICOS, PE, rat, IgG2b, k, 7E.17G9, BioLegendTM,100, 117406
IFN-ɣ, PE-Cy7, rat, IgG1, XMG1.2, eBioscience, 400, 25-7311-82
IFN-ɣ, APC, rat, IgG1, XMG1.2, BioLegendTM, 400, 505810
IL10 ,PE-Dazzle594, ra,t IgG2,b JES5-16E3, BioLegend, 100, 505033
KI-67, BV480, mouse, IgG1, B56, BD PharmigenTM, 100, 566109
KLRG-1, APC-C7, syrian hamster, IgG, 2F1/KLRG1, BioLegendTM, 100, 138426
LAG3, BV421, rat, IgG1, C9B7W, BioLegendTM, 100, 125221
Ly-6C, BV711, rat, IgG2c, HK1.4, BioLegendTM, 400, 128037
Ly6G, BUV563,rat, IgG2a, 1A8, BD PharmigenTM, 200, 565707
MerTK, PE-Cy7 , rat, IgG2a, DS5MMER, eBioscience, 100, 25-5751-82
MHC-II, BB700 rat, IgG2b, M5/114.15.2, BD, 400, 746197
NK1.1, BV785, mouse, gG2a, PK136, BioLegendTM, 100, 108749
OX40, Biotin, rat, IgG1, OX-86, BioLegendTM, 100, 119403
PD-1, BV785, rat, IgG2a, 29F.1A12, BioLegendTM, 100, 135225
PD-1, BV605, rat, IgG2a, 29F.1A12, BioLegendTM, 100, 135220
PD-L1, APC, rat, IgG2b, 10F.9G2, BioLegendTM, 150, 124311
PD-L1, PE-Cy7, rat, IgG2a, MIH5, eBioscience, 200, 25-5982-82
PD-L2, Dazle, rat, IgG2a, TY25 , BioLegendTM,150, 107216, 150, 107216
Streptavidin, BV570, BioLegend, 400, 405227
Streptavidin, BUV395, BD PharmigenTM, 400, 564176
TCRb, PE-Cy5, armenian hamster, IgG, H57-597, BioLegendTM, 400, 109209
TNF, BV711, rat, IgG, MP6-XT22, BioLegendTM, 200, 506349
XCR-1 Alexa647 mouse IgG2b ZET BioLegendTM, 200, 148213
mIgG2a (secondary Ab) PE Goat polyclonal Jackson 1000 115-115-206-JIR
EGFRvIII, Purified, Mouse IgG1 L84A Absolute Antibody 1000 Ab00184-1.1
CD34, Purified, Rat IgG2A, k, RAM34, eBioscience, 100 14-0341-82

Validation

All antibodies used are standard antibodies and have been validated by the manufacturers. All the information is available in the website of Biolgend (https://www.biolegend.com/en-us/reproducibility) and eBioscience and Thermofisher (https://www.thermofisher.com/ch/en/home/life-science/antibodies/invitrogen-antibody-validation.html). They were then titrated in house

in a total volume of 50 microliter on 1 milion of splenocytes or on cells derived from a specific organ for optimal use for flow cytometry.

Eukaryotic cell lines

Policy information about cell lines

Cell line source(s)	GL261 were provided by A. Fontana, Experimental Immunology, University of Zurich, Zurich, Switzerland. B16.F10 were bought from ATCC (CRL-6475™).
Authentication	GL261 were not authenticated. B16.F1O authentication was provided by ATCC.
Mycoplasma contamination	All cell lines tested negative for mycoplasma
Commonly misidentified lines (See ICLAC register)	No commonly misidentified lines from the ICLAC register were used in this study

Animals and other organisms

Policy information about studies involving animals; ARRIVE guidelines recommended for reporting animal research

Laboratory animals	Wild-type C57Bl/6J wild type mice (strain code 632) were purchased from Charles River and C57Bl/6 CD45.1 mice (strain code 494) were a kind gift of Sergio Quezada and were bred at Charles River. Female mice of 6 to 8 weeks of age were used in all experiments
Wild animals	No wild animals were used in this study
Field-collected samples	No field-collected samples were used in this study
Ethics oversight	All animal studies were performed under the approval of the University College London and U.K. Home Office and the Swiss Cantonal Veterinary Office Zurich.

Note that full information on the approval of the study protocol must also be provided in the manuscript.

Flow Cytometry

Plots

Confirm that:

☒ The axis labels state the marker and fluorochrome used (e.g. CD4-FITC).

☒ The axis scales are clearly visible. Include numbers along axes only for bottom left plot of group (a 'group' is an analysis of identical markers).

☒ All plots are contour plots with outliers or pseudocolor plots.

☒ A numerical value for number of cells or percentage (with statistics) is provided.

Methodology

Sample preparation	Brains were cut in small pieces and incubated with collagenase type IV, and DNase I for 30 minutes in Hank's Balanced Salt, followed by homogenization through needle. TILs were enriched by resuspension in Percoll (gradient 30%) and centrifugated. Cells were then washed twice, blocked using anti-CD32/CD16 to avoid non-specific binding and stained. Spleens were digested as described for the tumors. Erythrocytes were lysed using RBC lysis buffer. The cells were then spun and resuspended in PBS. Single-cell suspensions were directly used for staining. For re-stimulation, cells were incubated for 4 h at 37°C and 5% CO2 in re-stimulation medium. Prior to intracellular staining cells were fixed and permeabilized with fixation permeabilization solution according to manufacturer's instructions.
Instrument	After surface and intracellular staining, cells were acquired on a Symphony Flow Cytometer (BD Biosciences) or CyTEK Aurora flow cytometer (Cytek Biosciences).
Software	Data from Symphony were collected with FACS Diva Software (version 9, BD Biosciences). Data from Aurora were collected with SpectroFlo Software. FCS files were then exported and analyzed using FlowJo software (V9 or V10), Graphpad Prism (v6-8) and R studio Version 3.6.1.
Cell population abundance	It is expressed as frequency of the selected population
Gating strategy	Lymphocytes were defined by size and granularity in FSC-A vs SSC-A plots. Next, duplets were excluded in FSC-A vs FSC-H plots and dead cells were excluded by means of fixable viability dye positivity. T cells were gated as CD45highCD11b- and CD11b myeloid cells were gated as CD45highCD11b+ of living single cells in the brain and spleen samples.<br>Boundaries between positive and negative cells were defined by use of fluorescence minus one (FMO) controls. Expression of cytokines or surface markers was determined by calculating the median or mean fluorescence intensity. Gating strategies are shown in the supplementary figures.

☒ Tick this box to confirm that a figure exemplifying the gating strategy is provided in the Supplementary Information.

Magnetic resonance imaging

Experimental design

Design type	Block design. All animals within one experiment were subjected to MRI on the same day.
Design specifications	MRI was performed prior to treatment administration and then weekly until humane endpoints were reached.
Behavioral performance measures	No behavioral performance measures were performed as they were not relevant to this study.

Acquisition

Imaging type(s)	Structural T2-weighted
Field strength	1 Tesla
Sequence & imaging parameters	Images were acquired on a small animal 1T MRI scanner (Bruker ICON) with a 26 mm diameter mouse head coil. Images were acquired using a 2D T2–weighted sequence (TR = 3202ms, TE = 85ms, resolution = 0.21x0.21x0.50 mm 3, averages = 15; acquisition time of 6 minutes), axial orientation. Software version was Paravision 5 (Bruker BioSpin Gmbh, Ettlingen, Germany)
Area of acquisition	Whole brain

Diffusion MRI ☐ Used ☒ Not used

Preprocessing

Preprocessing software	T2-weighted images were reconstructed using vendor-default processing, then exported to ITK-Snap for manual segmentation. ROIs were manually delineated for every slice to define the tumor area. Tumor volumes were exported to Excel.
Normalization	No normalization was performed as no contrast agent was administered
Normalization template	No normalization template was used
Noise and artifact removal	Artifact or noise removal was not performed
Volume censoring	No censoring was performed. ITK SNAP was used to measure tumor volumes. Tumor volumes were exported to Excel.

Statistical modeling & inference

Model type and settings	No models were used in this study
Effect(s) tested	No effects were tested

Specify type of analysis: ☐ Whole brain ☒ ROI-based ☐ Both

Anatomical location(s): Specific type of analysis: N/A

Statistic type for inference (See Eklund et al. 2016)	N/A
Correction	N/A

Models & analysis

n/a	Involved in the study
☒	☐ Functional and/or effective connectivity
☒	☐ Graph analysis
☒	☐ Multivariate modeling or predictive analysis
